# Research on the Four-Component Borehole Strain Response to Rock Fracture

**DOI:** 10.3390/s26041302

**Published:** 2026-02-17

**Authors:** Yifan Li, Yongxing Shen, Zengchao Feng

**Affiliations:** Key Laboratory of In-Situ Property Improving, Mining of Ministry of Education, Taiyuan University of Technology, Taiyuan 030024, China; lifan202601@163.com (Y.L.); syx754578945@163.com (Y.S.)

**Keywords:** rock fracturing, four-component borehole strain gauge, strain response, strain response indices

## Abstract

Rock fracture monitoring is crucial for the stability of rock engineering. Based on the four-component borehole strain (FCBS) theory, this study analyzes the response characteristics of FCBS through numerical simulations of large-scale local rock fracture. Drawing on linear elastic mechanics theory and combined with the Gaussian white noise model, three strain response indices (areal strain index $p_{j}^{a}$ and shear strain indices $p_{j}^{13}$, $p_{j}^{24}$) are proposed to quantitatively characterize rock fracture events. A criterion is defined that if any of these indices is greater than 1, the rock fracture event can be reflected, and the larger the index, the better the effect of this index in reflecting rock fracture. The effects of the installation angle of the four-component borehole strain gauge (FCBSG), the distance between the borehole and the fracture zone, and the orientation of the borehole on these three indices are systematically investigated. The results show that for the same borehole, the areal strain index remains constant for different installation angles of the FCBSG, while the two shear strain indices exhibit a complementary variation trend—one shear strain index is always greater than or equal to the characteristic value of the borehole shear strain index, and the other is less than or equal to it; the larger values of the areal strain index and shear strain index decrease with the increase in the distance between the borehole and the fracture zone, following the variation law of the function y = ax^b^ with a negative exponent; there are significant differences in the larger values of the areal strain index and shear strain index among different orientation of the borehole, while those in the same orientation of the borehole relative to the fault fractured zone show a certain degree of complementarity, and the combined use of shear strain indices and areal strain index can better reflect rock fracture events; within the range of orientation of the borehole *β* = 0° to *β* = 90°, the minimum range of rock fracture that can be reflected by the three strain response indices is 55 m, the maximum range is 65 m, and the average range is 60.7 m.

## 1. Introduction

Rock, as a very common material in engineering [1], is involved in many engineering geological disasters, and research on rock fracture is crucial for understanding numerous engineering disasters.

In the field of rock fracture, there are various methods to characterize rock fracture: Zeng et al. [2] investigated the multi-frequency acoustic emission characteristics of critical fracture of coarse sandstone under uniaxial compression. Nicolas et al. [3] and Su et al. [4] studied the fracture behavior of rocks (such as granite, sandstone, and cement mortar) induced by local dynamic disturbances under true triaxial compression through acoustic emission signals. Huang et al. [5] used the acoustic emission method to analyze the mechanical properties, acoustic emission evolution law, fracture precursor characteristics, and fracture mechanism of red sandstone samples with different hole shapes. Song et al. [6] studied the dynamic fracture process of rock using the digital speckle method combined with a high-speed camera. Ma et al. [7] investigated the strain field during rock fracture using the digital speckle method. Peng et al. [8] studied the influence of the dip angle of intermediate fractures on the propagation and connection of cracks in fractured limestone using the three-dimensional digital image method. Xiao et al. [9] determined the overall damage and surface damage variables of coal samples and their evolution laws through cumulative acoustic emission energy counts and infrared radiation energy, respectively. Liu et al. [10] analyzed the fracture mode and damage evolution of rock with pre-set circular holes during rockbursts by studying the characteristics of acoustic emission (AE) and infrared radiation temperature (IRT). Zhou et al. [11] studied the evolution of fracture networks in coal during loading using X-ray micro-CT equipped with a uniaxial compression device. Li Y et al. [12] carried out three-dimensional visualization and quantitative characterization of the dynamic evolution of fractures in anthracite under uniaxial and triaxial compression using X-ray micro-CT equipment equipped with a triaxial compression device.

Borehole stress monitoring [13,14,15] and borehole strain monitoring [16,17,18,19,20,21,22,23,24,25] are suitable for long-term in situ monitoring. Among them, borehole strain monitoring enables a more direct and continuous assessment of the underground stress and deformation state [16,17], and is particularly applicable to the long-term monitoring requirements in mining and environmental management [18,19]. It plays an irreplaceable key role in fields such as tectonic movement research [20,21], earthquake precursor capture [22,23], in situ stress periodic variation monitoring [24], and mine disaster prevention and control [25]. However, there are few studies on the quantitative characterization of rock fracturing using four-component borehole strain (FCBS) at present.

Therefore, this study adopts numerical simulation methods to design a large-scale local rock fracture scenario and arranges boreholes at positions far from the fracture zone. Rock fracture has various forms, most of which are caused by crack propagation. Therefore, the failure of finite element elements caused by the propagation of a single crack is used to represent rock fracture, and the FCBS response characteristics during rock fracture are obtained. Combined with linear elastic mechanics, Gaussian noise is added to the strain of each component, and strain response indices are proposed to describe the strain response characteristics. Furthermore, the influence laws of the installation angle of the FCBSG, the distance between the borehole and the fracture zone, and the orientation of the borehole on the strain response indices are studied.

## 2. Establishment of the Numerical Simulation Model for Rock Fracture

### 2.1. Measurement Principle of the FCBSG

**Figure 1:**
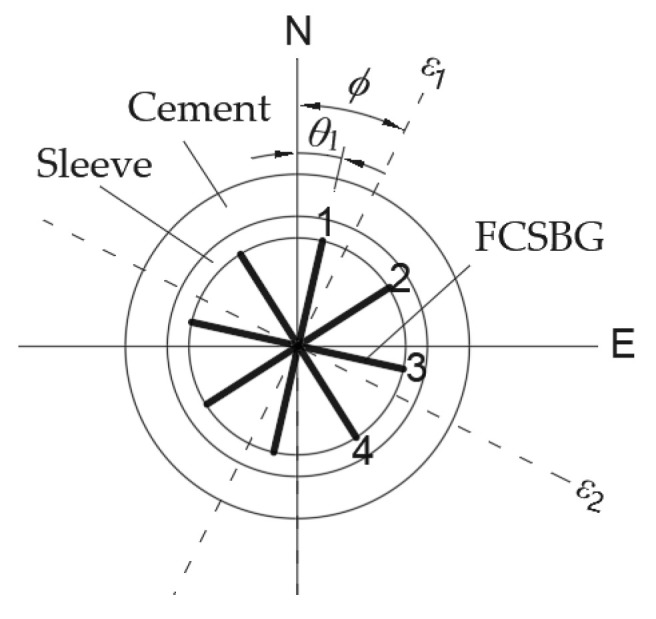
Strain tensor model for observation plane of FCBSG.

The probe of the borehole strainmeter adopts a cylindrical sealed sleeve structure, with sensors integrated inside for monitoring borehole diameter deformation. After completing the drilling operation, the probe is lowered into the borehole, and then special coupling cement is poured into the gap between the probe and the surrounding media, such as rock and soil. Effective observation can only be carried out after the coupling effect is fully achieved. As shown in Figure 1, the FCBSG is equipped with 4 strain sensors, which are placed radially at intervals of 45° in the borehole in a “meter” shape to record the relative radial changes in four directions. The relative change in inner diameter measured by the strain sensor in direction *θ* is [26]:

(1) $$S_{\theta }=A(\varepsilon_{1}+\varepsilon_{2})+B(\varepsilon_{1}-\varepsilon_{2})\cos(2\theta -2\phi )$$

where *ε*_1_ and *ε*_2_ are the maximum and minimum far-field horizontal principal strains, respectively; A and B are coupling coefficients related to the dimensions and materials of the cement and sleeve, as well as the surrounding lithology; *ϕ* is the direction of *ε*_1_.

Let the direction of the first strain sensor be *θ*_1_. The strains measured by the four strain sensors are [27]:

(2) $$\left\{\begin{array}{c} S_{1}=S_{\theta_{1}}=A(\varepsilon_{1}+\varepsilon_{2})+B(\varepsilon_{1}-\varepsilon_{2})\cos(2\theta_{1}-2\phi )\\ S_{2}=S_{\theta_{1}+\pi /4}=A(\varepsilon_{1}+\varepsilon_{2})-B(\varepsilon_{1}-\varepsilon_{2})\sin(2\theta_{1}-2\phi )\\ S_{3}=S_{\theta_{1}+\pi /2}=A(\varepsilon_{1}+\varepsilon_{2})-B(\varepsilon_{1}-\varepsilon_{2})\cos(2\theta_{1}-2\phi )\\ S_{4}=S_{\theta_{1}+3\pi /4}=A(\varepsilon_{1}+\varepsilon_{2})+B(\varepsilon_{1}-\varepsilon_{2})\sin(2\theta_{1}-2\phi ) \end{array}\right.$$

Since there are three independent parameters in the plane strain state, the four direct observations are converted into three surrogate observations [28]:

(3) $$\left\{\begin{array}{c} S_{a}=(S_{1}+S_{2}+S_{3}+S_{4})/2\\ S_{13}=S_{1}-S_{3}\\ S_{24}=S_{2}-S_{4} \end{array}\right.$$

where *S*_a_ denotes the areal strain, and *S*_13_ and *S*_24_ are two independent shear strains. Substituting Equation (2) into Equation (3) yields the surrogate observations [28]:

(4) $$\left\{\begin{array}{c} S_{a}=2A(\varepsilon_{1}+\varepsilon_{2})\\ S_{13}=2B(\varepsilon_{1}-\varepsilon_{2})\cos(2\theta_{1}-2\phi )\\ S_{24}=-2B(\varepsilon_{1}-\varepsilon_{2})\sin(2\theta_{1}-2\phi ) \end{array}\right.$$

The three sets of sequences (*S*_a_, *S*_13_, and *S*_24_) are often used as important data for studying earthquake precursors and occurrences. For example, Qiu Zehua et al. [29] extracted abnormal changes before the Wenchuan Earthquake by conducting wavelet analysis on these three sequences; Zhu Kaiguang et al. [30] proposed a method for processing the data of *S*_a_, *S*_13_, and *S*_24_ sequences and then using principal component analysis to extract abnormal signals; Chi Chengquan [31] converted borehole strain data into these three sequences for further anomaly extraction. This study also characterizes rock fracture based on these three sequences.

### 2.2. Principle of Numerical Simulation for Rock Fracture

The cohesive zone model (CZM) has been widely applied in geotechnical engineering fields such as brittle rock fracturing [32], slope stability [33], mining engineering [34], and tunnel engineering [35]. Based on the concept of damage evolution around discontinuous surfaces (pre-existing defects) after crack initiation, this model characterizes the mechanical behavior of discontinuous surfaces through the traction–separation law. In this study, the CZM is combined with the extended finite element method (XFEM), and numerical simulations are carried out via the finite element analysis software ABAQUS. The widely used maximum principal stress criterion is adopted to describe the initiation conditions of crack nucleation.

(5) $$\langle \sigma_{\max}\rangle =\frac{1}{2}(\sigma_{\max}+|\sigma_{\max}|)$$

(6) $$f=\left\{\frac{\langle \sigma_{\max}\rangle }{\sigma_{\max}^{o}}\right\}$$

where $\sigma_{\text{max}}^{o}$ denotes the maximum allowable principal stress; here, the Macaulay bracket is used to indicate that pure compressive stress or strain states will not cause damage. When the maximum principal stress ratio *f* reaches 1 and falls within the following range, cracks begin to form and propagate:

(7) $$1.0\leq f\leq 1.0+f_{\text{def}}$$

where *f*_def_ is the default error value, taken as 0.05. If (*f* > 1 + *f*_def_), the time increment will be reduced until the crack initiation criterion is satisfied.

**Figure 2:**
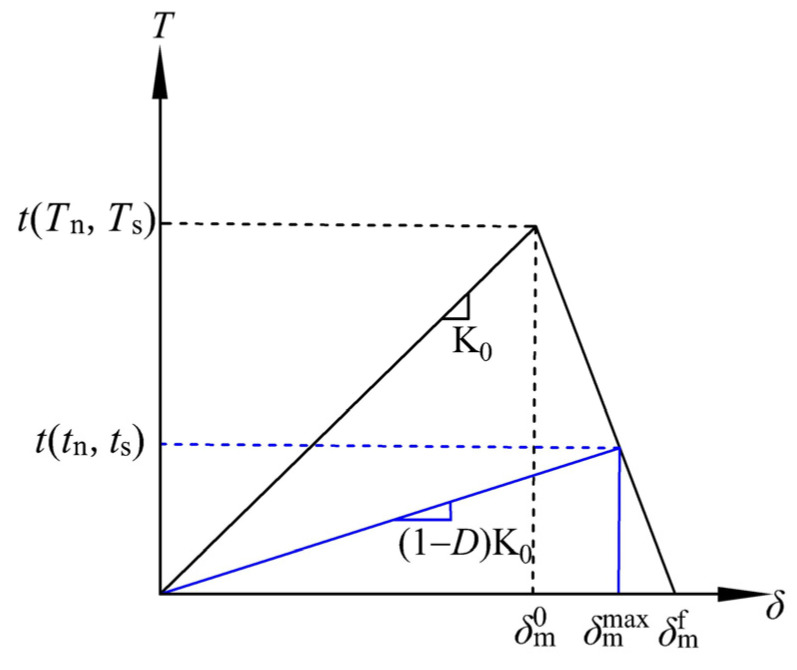
Traction–separation law.

The traction–separation law adopted in this study is shown in Figure 2: When a load is applied, the traction components acting on the defect gradually increase until reaching the tensile and shear limits of the material. Once the load exceeds the material limits, new cracks initiate, and the traction components at the defect tip decrease accordingly. The traction stress is expressed as:

(8) $$T_{n}=K_{0}\delta_{n}$$

(9) $$t_{n}=(1-D)T_{n}$$

(10) $$T_{s}=K_{0}\delta_{s}$$

(11) $$t_{s}=(1-D)T_{s}$$

where *t*_n_ and *t*_s_ are the instantaneous tensile and shear traction stresses, respectively; *T*_n_ and *T*_s_ are the tensile and shear traction stresses predicted by elastic traction–separation behavior for the current separation without damage, respectively; *δ*_n_ and *δ*_s_ are the relative displacements at the prefabricated defect caused by tensile and shear traction stresses, respectively; K_0_ is the stiffness of the cohesive model without damage; and *D* is the stiffness damage factor.

Initially, *D* = 0, indicating that the material is undamaged and remains in the elastic state. After damage initiation, the stiffness damage factor *D* increases until *D* = 1, at which point the material completely fails. In Abaqus, the cohesive stiffness damage factor *D* is used, and its expression is:

(12) $$D=\frac{\delta_{m}^{f}(\delta_{m}^{\text{max}}-\delta_{m}^{0})}{\delta_{m}^{\text{max}}(\delta_{m}^{f}-\delta_{m}^{0})}$$

where $\delta_{m}^{\text{max}}$ is the maximum effective displacement during the interface damage evolution, $\delta_{n}^{0}$ is the effective displacement at the onset of interface damage, and $\delta_{m}^{f}$ is the effective displacement at complete interface failure.

When $\delta_{m}^{\text{max}}$ reaches $\delta_{m}^{0}$, new cracks start to initiate, and *D* = 0 at this moment; when $\delta_{m}^{0}$ < $\delta_{m}^{\text{max}}$ < $\delta_{m}^{f}$, the initiated cracks continue to propagate, and *D* linearly increases from 0 to 1; when $\delta_{m}^{\text{max}}$ > $\delta_{m}^{f}$, the material reaches a state of complete damage, and *D* = 1. To describe the damage evolution under the combination of normal and shear deformations at the interface for relative displacement in mixed modes, the effective displacement is introduced:

(13) $$\delta_{m}=\sqrt{{\langle \delta_{n}\rangle }^{2}+\delta_{s}^{2}}$$

### 2.3. Establishment of the Numerical Simulation Model

**Figure 3:**
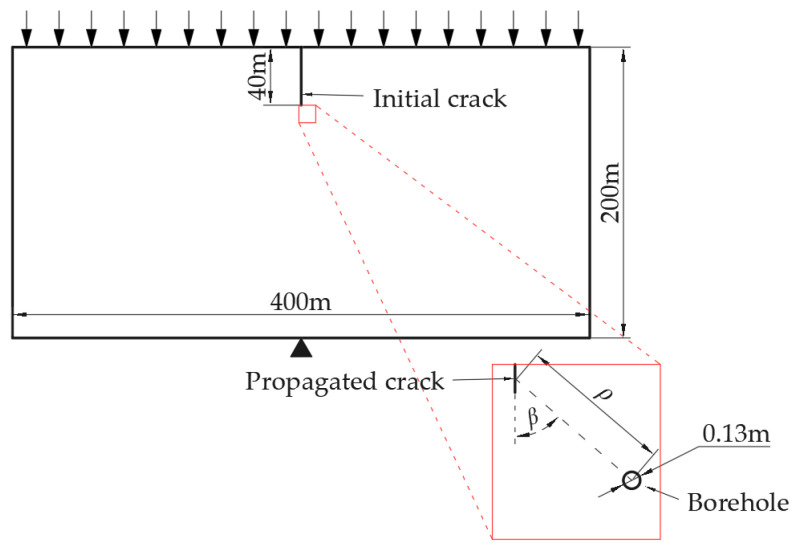
Schematic diagram of model.

To characterize rock fracture using FCBS, it is necessary to avoid the borehole becoming part of the rock fracture zone. In this study, a large-scale rock model is designed through numerical simulation, as shown in Figure 3. Initial cracks are designed to control the position of propagating cracks, and boreholes are placed far from the propagating cracks to ensure the integrity of the rock around the boreholes. A fixed constraint is set at the midpoint of the bottom of the model, and uniform loads are applied to the top to induce crack propagation under slow and constant loading conditions, thereby studying the FCBS response characteristics.

**Figure 4:**
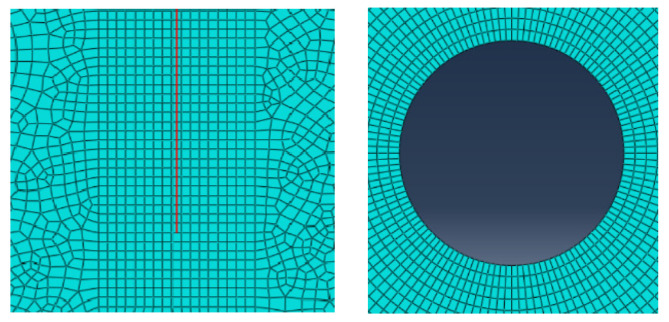
Mesh generation around crack tips and boreholes.

By controlling the direction of propagating cracks to be consistent with that of initial cracks, crack turning is avoided, which would otherwise lead to inaccurate stress fields around the turning points due to insufficient mesh division. The mesh division of cracks and the borehole in this numerical simulation is shown in Figure 4.

The mesh generation around the cracks and boreholes in the numerical simulation is shown in Figure 4. The mesh around the cracks is square and parallel to the cracks. Due to the geometric symmetry of the model and the symmetry of the load constraints, under the action of uniform loads parallel to the cracks, the rock bends and deforms to the left and right sides, and the newly formed propagating cracks will be consistent with the direction of the initial cracks. This avoids inaccurate stress fields around the crack turning points caused by insufficient mesh generation at the turning points. Utilizing the characteristic of XFEM in Abaqus that crack tips can only appear on mesh boundaries, the length of each propagating crack can be ensured to be consistent, thereby controlling variables.

Under slow and constant loading conditions, to study the characteristics of rock fracture compared to the elastic stage, it is necessary to ensure a sufficiently long elastic stage, while the fracture stage only consists of 1 loading step. Thus, the length of the initial crack is designed to be much larger than that of the propagating crack. The initial crack length is set to 40 m, and the length of the propagating crack is controlled to 0.1 m through mesh generation. Large-scale sandstone formations often contain natural fractures [36,37] and mining-induced fractures [38,39] with scales of tens to even hundreds of meters. The mechanical parameters of sandstone adopted in this paper [40,41,42,43] are listed in Table 1. The load is increased by 40 Pa in each loading step until crack propagation occurs. The number of loading steps for all numerical simulations when cracks propagate is 204, with the initial crack of 40 m extending to 40.1 m (a propagation length of 0.1 m), causing the failure of one finite element, which is defined as the rock fracture zone.

### 2.4. Validation of Numerical Simulation Results

**Figure 5:**
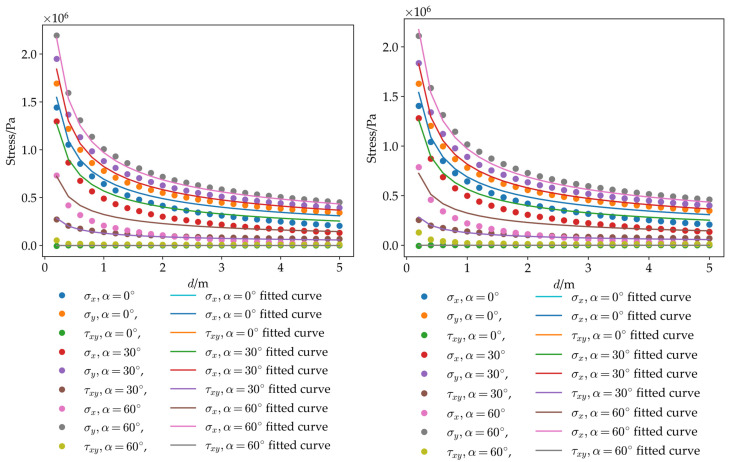
The numerically simulated stress values and the fitted stress values.

To verify the correctness of the numerical results obtained in this study, the stress formula for the vicinity of the crack tip was adopted for validation. A local coordinate system was established at the crack tip, where the crack tip was set as the pole, and the crack propagation direction was defined as the polar axis pointing to the crack-free end. The coordinates of any arbitrary point in this local system were expressed as (*d*,*α*). Stress values along the directions of *α* = 0°, *α* = 30°, and *α* = 60° near the crack tip were fitted using the crack-tip stress formula. The comparison curves between the numerically simulated stress values and the fitted stress values are plotted in Figure 5. The favorable fitting effect demonstrates the correctness of the numerical simulation.

## 3. Strain Response Characteristics and Strain Response Indices

### 3.1. FCBS Response Characteristics of Rock Fracture

**Figure 6:**
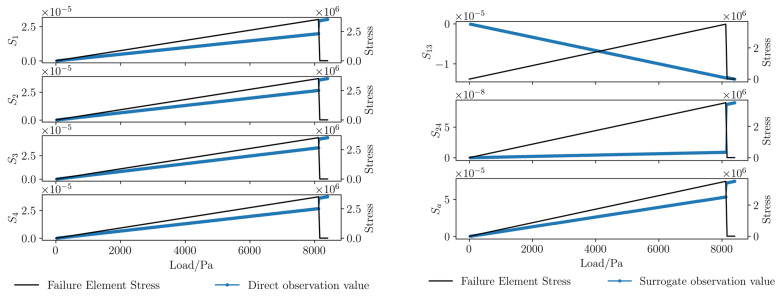
Curves of direct observations and surrogate observations in numerical simulation.

In this study, the applied load is taken as the abscissa, and the stress state of the crack-propagating element is taken as the ordinate to characterize rock fracture. Through the analysis of the data around the boreholes from the numerical simulation results, curves of direct observation values and surrogate observation values are plotted, as shown in Figure 6. Before element fracture, the direct observations and surrogate observations change linearly with the uniform increase in the load; at the moment of element fracture, some direct observations and surrogate observations undergo sudden changes; after element fracture, the direct observations and surrogate observations resume linear changes.

**Figure 7:**
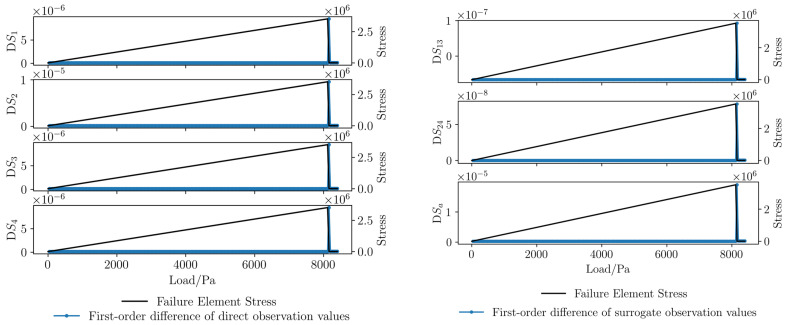
Curves of first-order differences for direct observations and surrogate observations in numerical simulation.

First-order difference curves of direct observations and surrogate observations are plotted, as shown in Figure 7 (corresponding to the curves in Figure 6): Before element fracture, the first-order differences of direct observations and surrogate observations remain basically constant with the uniform increase in the load; at the moment of element fracture, the first-order differences of direct observations and surrogate observations undergo sudden changes; after element fracture, the first-order differences of direct observations and surrogate observations resume a basically constant state.

Comparing Figure 6 and Figure 7, it can be seen that the sudden changes in some strain sequences are not obvious, but the sudden changes in strain difference sequences are more significant. The reason is that in the elastic stage, the first-order strain difference represents the strain change caused by load increase; at the moment of fracture, the first-order strain difference represents the strain change caused by the coupling effect of load increase and rock fracture. Therefore, the first-order difference can better reflect the characteristics of rock fracture.

### 3.2. FCBS Response Indices of Rock Fracture

**Figure 8:**
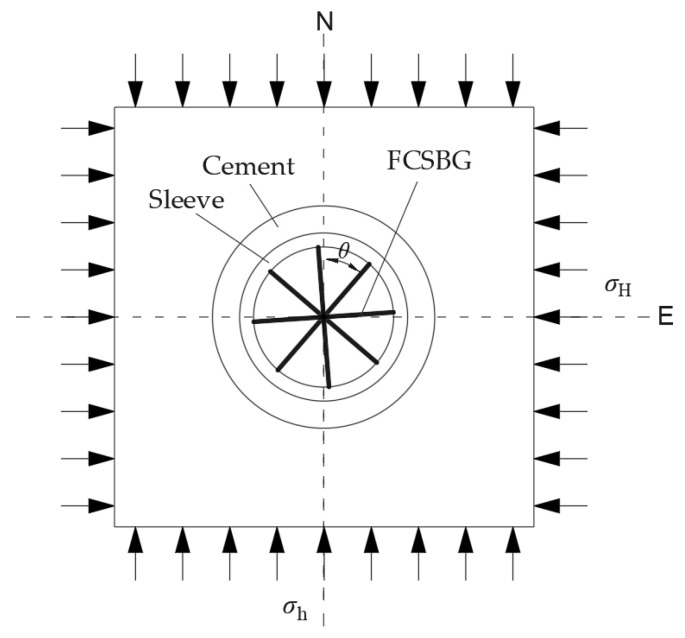
Elastomer model with FCBSG under steady loading conditions.

To describe this response characteristic, strain response indices are established in this study based on linear elastic mechanics combined with an error model. As shown in Figure 8, consider a linear elastic model equipped with an FCBSG. In the initial state, the elastic body is subjected to external loads at a location far from the borehole. The system adopts a steady loading mode, where all loads increase synchronously at a constant rate. During this period, strain data from the four strain sensors of the FCBSG are recorded at equal time intervals.

The strain measured by the strain sensor at direction *θ* at time *j* is denoted as *ε_θ__,j_*, and the first-order difference in the strain at time *j* for the strain sensor at direction *θ* is denoted as *δ_θ,j_*, i.e.,

(14) $$\delta_{\theta ,j}=\varepsilon_{\theta ,j}-\varepsilon_{\theta ,j-1}$$

Further, the first-order differences of the surrogate observations are obtained:

(15) $$\left\{\begin{array}{c} \delta_{a,j}=(\delta_{\theta_{1},j}+\delta_{\theta_{1}+\pi /4,j}+\delta_{\theta_{1}+\pi /2,j}+\delta_{\theta_{1}+3\pi /4,j})/2\\ \delta_{13,j}=\delta_{\theta_{1},j}-\delta_{\theta_{1}+\pi /2,j}\\ \delta_{24,j}=\delta_{\theta_{1}+\pi /4,j}-\delta_{\theta_{1}+3\pi /4,j} \end{array}\right.$$

Assume that the elastic body does not fracture from time a to time b but fractures at time b + 1. Since all loads are assumed to increase uniformly, according to linear elastic mechanics, the strain sequences obtained by the strain sensors in each direction from time a to time b are linear functions of time, and the first-order strain difference sequences are constant sequences, denoted as:

(16) $$\delta_{\theta ,j}=k_{\theta },(a+1\leq j\leq b)$$

Similarly, the difference sequences of the surrogate observations further calculated are also constant sequences, denoted as:

(17) $$\left\{\begin{array}{c} \delta_{a,j}=k_{a}\\ \delta_{13,j}=k_{13}\\ \delta_{24,j}=k_{24} \end{array}\right.$$

**Figure 9:**
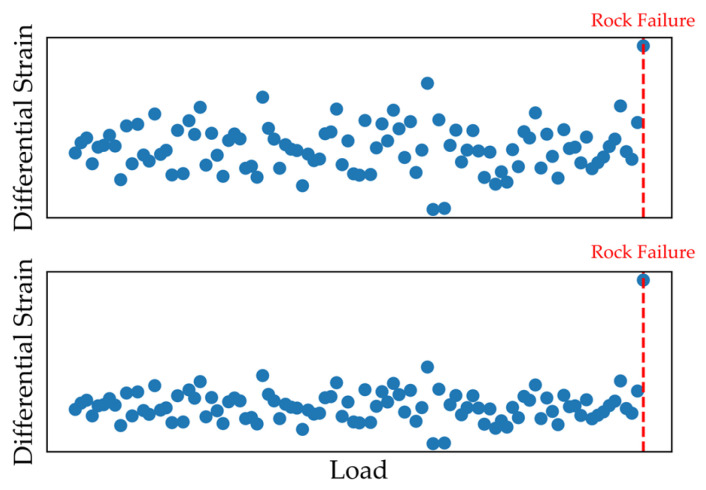
Schematic diagram of different deviation degrees of differential strain in rock failure.

At time b + 1, the elastic body fractures, and the first-order differences deviate from those from time a + 1 to time b. Thus, the difference between them can be used to characterize the response characteristics during fracture. However, considering practical conditions, due to various factors, the difference sequences in the elastic stage before fracture are not completely equal, as shown in Figure 9. Whether the response characteristic of the differential strain sequence during rock fracture is obvious is related to the fluctuation range of the differential strain sequence before rock fracture and the deviation degree of the differential strain sequence during rock fracture. Therefore, the influence of error should be considered, and an error model should be established.

Yu et al. [44] performed the Kolmogorov–Smirnov (K-S) test on the first-order difference in strain data after removing environmental strain responses, verifying that the daily crustal strain signals are approximately Gaussian distributed when free from earthquakes and heavy rainfall. This is consistent with the hypothesis proposed by Sun et al. [45] that short-period high-frequency signals follow a normal distribution in the absence of other disturbances. Similarly, Langbein [46] demonstrated that most noise in borehole strain measurements conforms to a Gaussian distribution. In this study, Gaussian noise with the same distribution is added to the first-order difference sequences obtained by each strain sensor based on linear elastic mechanics. If the difference between the first-order strain difference after adding Gaussian noise and the first-order strain difference considering only linear elastic theory for the strain sensor at direction *θ* at time j is *e_θ,j_*, then:

(18) $$\delta_{\theta ,j}^{c}=\delta_{\theta ,j}+e_{\theta ,j}$$

Then the first-order difference in the surrogate observations is:

(19) $$\left\{\begin{array}{c} \delta_{a,j}^{c}=\delta_{a,j}+(e_{\theta_{1},j}+e_{\theta_{1}+\pi /4,j}+e_{\theta_{1}+\pi /2,j}+e_{\theta_{1}+3\pi /4,j})/2\\ \delta_{13,j}^{c}=\delta_{13,j}+e_{\theta_{1},j}-e_{\theta_{1}+\pi /2,j}\\ \delta_{24,j}^{c}=\delta_{24,j}+e_{\theta_{1}+\pi /4,j}-e_{\theta_{1}+3\pi /4,j} \end{array}\right.$$

Let

(20) $$\left\{\begin{array}{c} e_{a,j}=(e_{\theta_{1},j}+e_{\theta_{1}+\pi /4,j}+e_{\theta_{1}+\pi /2,j}+e_{\theta_{1}+3\pi /4,j})/2\\ e_{13,j}=e_{\theta_{1},j}-e_{\theta_{1}+\pi /2,j}\\ e_{24,j}=e_{\theta_{1}+\pi /4,j}-e_{\theta_{1}+3\pi /4,j} \end{array}\right.$$

Then Equation (19) can be rewritten as:

(21) $$\left\{\begin{array}{c} \delta_{a,j}^{c}=\delta_{a,j}+e_{a,j}\\ \delta_{13,j}^{c}=\delta_{13,j}+e_{13,j}\\ \delta_{24,j}^{c}=\delta_{24,j}+e_{24,j} \end{array}\right.$$

After Gaussian noise is taken into account, during the period from time a + 1 to time b:

(22) $$\delta_{\theta ,j}^{c}=k_{\theta }+e_{\theta ,j},(a+1\leq j\leq b)$$

Similarly, the first-order strain differences of the surrogate observations with Gaussian noise added during the period from time a + 1 to time b are:

(23) $$\left\{\begin{array}{c} \delta_{a,j}^{c}=k_{a}+e_{a,j}\\ \delta_{13,j}^{c}=k_{13}+e_{13,j}\\ \delta_{24,j}^{c}=k_{24}+e_{24,j} \end{array}\right.$$

To evaluate whether the difference sequence can reflect the occurrence of rock fracture, we use the hypothesis-testing method in probability statistics. We assume that the difference sequence cannot reflect rock failure events—that is, the mean value at time *j* = *b* + 1 is equal to the mean value over the period from *j* = *a* + 1 to *j* = *b*. Then:

(24) $$\left\{\begin{array}{c} \frac{1}{\sigma_{a}}\left|\delta_{a,j}^{c}-k_{a}\right|∼N(0,1)\\ \frac{1}{\sigma_{13}}\left|\delta_{13,j}^{c}-k_{13}\right|∼N(0,1)\\ \frac{1}{\sigma_{24}}\left|\delta_{24,j}^{c}-k_{24}\right|∼N(0,1) \end{array}\right.,(j=b+1)$$

In the formula, *σ*_a_, *σ*_13_, and *σ*_24_ denote the standard deviations of the first-order difference sequences of the three alternative observed values, respectively.

The null hypothesis is rejected in accordance with the 3σ principle. Thus, the following indicators can be used to characterize the response characteristics of four-component borehole strain to rock fracture:

(25) $$\left\{\begin{array}{c} p_{j}^{a}=\frac{1}{c_{a}}\left|\delta_{a,j}^{c}-k_{a}\right|\\ p_{j}^{13}=\frac{1}{c_{13}}\left|\delta_{13,j}^{c}-k_{13}\right|\\ p_{j}^{24}=\frac{1}{c_{24}}\left|\delta_{24,j}^{c}-k_{24}\right| \end{array}\right.,(j=b+1)$$

where c_a_, c_13_, and c_24_ are the Gaussian noise error ranges of the first-order difference sequences of the three alternative observed values, respectively; $p_{j}^{a}$ is the areal strain index; and $p_{j}^{13}$ and $p_{j}^{24}$ are the shear strain indices.

Since the same distribution of Gaussian noise is added to the difference sequence of each sensor, let its error range be c, then:

(26) $$\left\{\begin{array}{l} c_{a}=c\\ c_{13}=\sqrt{2}c\\ c_{24}=\sqrt{2}c \end{array}\right.$$

Substituting it into Equation (25) yields:

(27) $$\left\{\begin{array}{c} p_{j}^{a}=\frac{1}{c}\left|\delta_{a,j}^{c}-k_{a}\right|\\ p_{j}^{13}=\frac{1}{\sqrt{2}c}\left|\delta_{13,j}^{c}-k_{13}\right|\\ p_{j}^{24}=\frac{1}{\sqrt{2}c}\left|\delta_{24,j}^{c}-k_{24}\right| \end{array}\right.,(j=b+1)$$

If any of these indicators is greater than 1, the null hypothesis is rejected. It is assumed that the mean value at time *j* = *b* + 1 is not equal to the mean value from time *j* = *a* + 1 to *j* = *b;* that is, when rock fracture occurs at time *j* = *b* + 1, the strain response characteristics of the difference sequence of the corresponding alternative observed values are significant and can reflect the rock fracture event. The larger the indicator, the more pronounced the strain response characteristics.

## 4. Influences of Installation Angle, Distance, and Direction on Strain Response Indices

### 4.1. Influence Law of Installation Angle on Strain Response Indices

In plane stress problems, the linear strain of rock without holes is:

(28) $$\left\{\begin{array}{c} \varepsilon_{1}=(\sigma_{1}-\nu \sigma_{2})/E\\ \varepsilon_{2}=(\sigma_{2}-\nu \sigma_{1})/E \end{array}\right.$$

Substituting it into Equation (1):

(29) $$S_{\theta }=\frac{1-\nu }{E}A(\sigma_{1}+\sigma_{2})+\frac{1+\nu }{E}B(\sigma_{1}-\sigma_{2})\text{cos}(2\theta -2\phi )$$

Let

(30) $$\left\{\begin{array}{c} A_{1}=\frac{1-\nu }{E}A\\ B_{1}=\frac{1+\nu }{E}B \end{array}\right.$$

Equation (29) can be rewritten as:

(31) $$S_{\theta }=A_{1}(\sigma_{1}+\sigma_{2})+B_{1}(\sigma_{1}-\sigma_{2})\cos(2\theta -2\phi )$$

According to the stress state theory at a point in elastic mechanics, Equation (31) can be expressed as:

(32) $$S_{\theta }=A_{1}(\sigma_{N}+\sigma_{E})+B_{1}(\sigma_{N}-\sigma_{E})\text{cos}2\theta +2B_{1}\tau_{\text{NE}}\text{sin}2\theta$$

where *σ*_N_, *σ*_E_, and *τ*_NE_ are the normal stresses in the far-field north and east directions and the shear stress, respectively.

Thus, Equation (4) can be rewritten as

(33) $$\left\{\begin{array}{c} S_{a}=2A_{1}(\sigma_{N}+\sigma_{E})\\ S_{13}=2B_{1}(\sigma_{N}-\sigma_{E})\cos2\theta_{1}+4B_{1}\tau_{\text{NE}}\sin2\theta_{1}\\ S_{24}=-2B_{1}(\sigma_{N}-\sigma_{E})\sin2\theta_{1}+4B_{1}\tau_{\text{NE}}\cos2\theta_{1} \end{array}\right.$$

At time *j*, when the far-field north-direction in situ stress changes by *γ*_N,*j*_, the east-direction in situ stress changes by *γ*_E,*j*_, and the shear stress changes by *γ*_NE,*j*_, the change value of the strain sensor arranged at direction *θ* is:

(34) $$\delta_{j}=A_{1}(\gamma_{N,j}+\gamma_{E,j})+B_{1}(\gamma_{N,j}-\gamma_{E,j})\cos2\theta +2B_{1}\gamma_{\text{NE},j}\sin2\theta$$

At time *j*, the first-order difference in the surrogate observations of the FCBSG is:

(35) $$\left\{\begin{array}{c} \delta_{a,\;j}=2A_{1}(\gamma_{N,j}+\gamma_{E,j})\\ \delta_{13,j}=2B_{1}(\gamma_{N,j}-\gamma_{E,j})\cos2\theta_{1}+4B_{1}\gamma_{\text{NE},j}\sin2\theta_{1}\\ \delta_{24,j}=-2B_{1}(\gamma_{N,j}-\gamma_{E,j})\sin2\theta_{1}+4B_{1}\gamma_{\text{NE},j}\cos2\theta_{1} \end{array}\right.$$

We assume that the elastic body does not fracture from time a to time b but fractures at time b + 1. The far-field stress around the borehole increases uniformly from time a to time b. Let *γ*_N,*j*_ = k_N_, *γ*_E,*j*_ = k_E_, and *γ*_NE,*j*_ = k_NE_; the first-order differences of the surrogate observations from time a + 1 to time b are:

(36) $$\left\{\begin{array}{c} \delta_{a,\;j}=2A_{1}(k_{N}+k_{E})\\ \delta_{13,j}=2B_{1}(k_{N}-k_{E})\cos2\theta_{1}+4B_{1}k_{\text{NE}}\sin2\theta_{1}\\ \delta_{24,j}=-2B_{1}(k_{N}-k_{E})\sin2\theta_{1}+4B_{1}k_{\text{NE}}\cos2\theta_{1} \end{array}\right.$$

At *j* = b + 1, let *λ*_1_ = *γ*_N,*j*_ − k_N_, *λ*_2_ = *γ*_E,*j*_ − k_E_, and *λ*_3_ = *γ*_NE,*j*_ − k_NE_. According to Equation (26), the strain response indices are:

(37) $$\left\{\begin{array}{l} p_{j}^{a}=\frac{1}{c}\left|2A_{1}(\lambda_{1}+\lambda_{2})+e_{a,j}\right|\\ p_{j}^{13}=\frac{1}{\sqrt{2}c}\left|2B_{1}(\lambda_{1}-\lambda_{2})\cos2\theta_{1}+4B_{1}\lambda_{3}\sin2\theta_{1}+e_{13,j}\right|\\ p_{j}^{24}=\frac{1}{\sqrt{2}c}\left|-2B_{1}(\lambda_{1}-\lambda_{2})\sin2\theta_{1}+4B_{1}\lambda_{3}\cos2\theta_{1}+e_{24,j}\right| \end{array}\right.$$

Given that the deviation degree of the first-order difference in strain during rock fracturing is relatively large, and noise has almost no effect on this deviation degree, Equation (37) is thus simplified to:

(38) $$\left\{\begin{array}{c} p_{j}^{a}=\frac{2A_{1}}{c}\left|\left(\lambda_{1}+\lambda_{2}\right)\right|\\ p_{j}^{13}=\frac{\sqrt{2}B_{1}}{c}\left|(\lambda_{1}-\lambda_{2})\cos2\theta_{1}+2\lambda_{3}\sin2\theta_{1}\right|\\ p_{j}^{24}=\frac{\sqrt{2}B_{1}}{c}\left|-(\lambda_{1}-\lambda_{2})\sin2\theta_{1}+2\lambda_{3}\cos2\theta_{1}\right| \end{array}\right.$$

It can be seen that among these three indices, the areal strain index $p_{j}^{a}$ is independent of the installation angle of the FCBSG, while the shear strain indices $p_{j}^{13}$ and $p_{j}^{24}$ are related to the installation angle of the FCBSG. Let:

(39) $$\left\{\begin{array}{c} a=(\lambda_{1}-\lambda_{2},2\lambda_{3})\\ m_{1}=(\cos2\theta_{1},\sin2\theta_{1})\\ m_{2}=(-\sin2\theta_{1},\cos2\theta_{1}) \end{array}\right.$$

Then, the shear strain indices in Equation (38) can be rewritten as:

(40) $$\left\{\begin{array}{c} p_{j}^{13}=\frac{\sqrt{2}B_{1}}{c}\left|a_{j}\cdot m_{1}\right|\\ p_{j}^{24}=\frac{\sqrt{2}B_{1}}{c}\left|a_{j}\cdot m_{2}\right| \end{array}\right.$$

**Figure 10:**
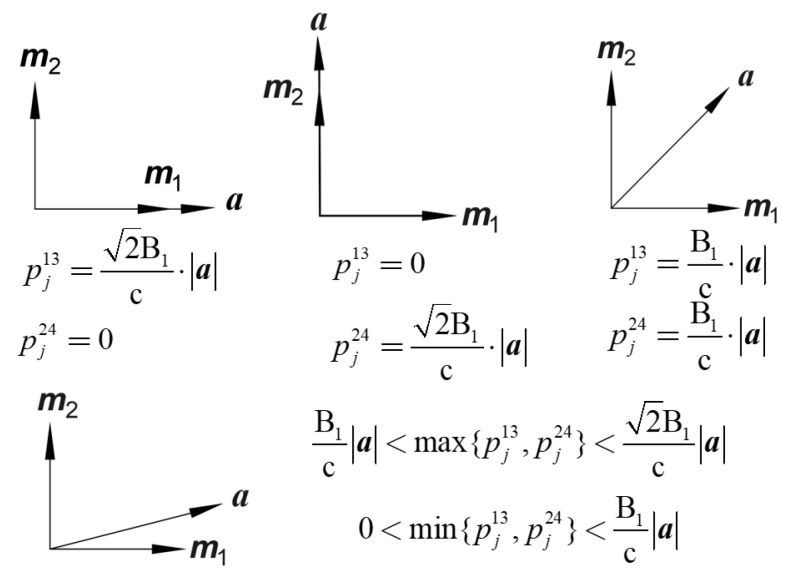
The shear strain sequence indices corresponding to different positional relationships between ***a*** and ***m***_1_, ***m***_2_.

Obviously, ***m***_1_ and ***m***_2_ are a set of perpendicular unit vectors, and ***a*** is a vector related to the change in the first-order strain difference. As shown in Figure 10, when ***a*** is parallel to ***m***_1_, $p_{j}^{13}$ has the best effect in reflecting rock fracture, and $p_{j}^{24}$ has the worst effect. At this time, the shear strain indices are:

(41) $$\left\{\begin{array}{l} p_{j}^{13}=\frac{\sqrt{2}B_{1}}{c}\cdot \left|a\right|=\frac{\sqrt{2}B_{1}}{c}\cdot \sqrt{{\left(\lambda_{1}-\lambda_{2}\right)}^{2}+4\lambda_{3}^{2}}\\ p_{j}^{24}=0 \end{array}\right.$$

When ***a*** is parallel to ***m***_2_, $p_{j}^{24}$ has the best effect in reflecting rock fracture, and $p_{j}^{13}$ has the worst effect. At this time, the shear strain indices are:

(42) $$\left\{\begin{array}{l} p_{j}^{13}=0\\ p_{j}^{24}=\frac{\sqrt{2}B_{1}}{c}\cdot \left|a\right|=\frac{\sqrt{2}B_{1}}{c}\cdot \sqrt{{\left(\lambda_{1}-\lambda_{2}\right)}^{2}+4\lambda_{3}^{2}} \end{array}\right.$$

When the angle between ***a*** and both ***m***_1_ and ***m***_2_ is 45°, $p_{j}^{13}$ and $p_{j}^{24}$ have the same effect in reflecting rock fracture, both equal to $\sqrt{2}/2$ times the maximum value:

(43) $$p_{j}^{13}=p_{j}^{24}=\frac{B_{1}}{c}\cdot \left|a\right|=\frac{B_{1}}{c}\cdot \sqrt{{\left(\lambda_{1}-\lambda_{2}\right)}^{2}+4\lambda_{3}^{2}}$$

For other positional relationships between ***a*** and ***m***_1_, ***m***_2_, the larger one of $p_{j}^{13}$ and $p_{j}^{24}$ is greater than the shear strain index when the angle is 45°, and the smaller one is less than the shear strain index when the angle is 45°, i.e.,

(44) $$\left\{\begin{array}{c} \frac{B_{1}}{c}\left|a\right|<\text{max}\{p_{j}^{13},p_{j}^{24}\}<\frac{\sqrt{2}B_{1}}{c}\left|a\right|\\ 0<\min\{p_{j}^{13},p_{j}^{24}\}<\frac{B_{1}}{c}\left|a\right| \end{array}\right.$$

In summary, regardless of the change in the installation direction of the FCBSG, one of the shear strain indices must be greater than or equal to the shear strain index when the angle is between ***a*** and ***m***_1_, ***m***_2_, i.e.,

(45) $$\max\{p_{j}^{13},p_{j}^{24}\}\geq \frac{B_{1}}{c}\left|a\right|$$

The shear strain index when the angle between ***a*** and ***m***_1_, ***m***_2_, is 45° is taken as the characteristic value of the shear strain index for different installation angles of the FCBSG in the same borehole, denoted as $p_{j}^{s}$, with the expression:

(46) $$p_{j}^{s}=\frac{B_{1}}{c}\left|a\right|=\frac{B_{1}}{c}\cdot \sqrt{{\left(\lambda_{1}-\lambda_{2}\right)}^{2}+4\lambda_{3}^{2}}$$

$p_{j}^{s}$ > 1 indicates that in the same borehole, regardless of the installation angle of the FCBSG, the larger shear strain index of the two shear strain indices is greater than 1, i.e., the larger shear strain index can reflect the rock fracture event.

### 4.2. Verification of the Influence Law of the Installation Angle on Strain Response Indices

**Figure 11:**
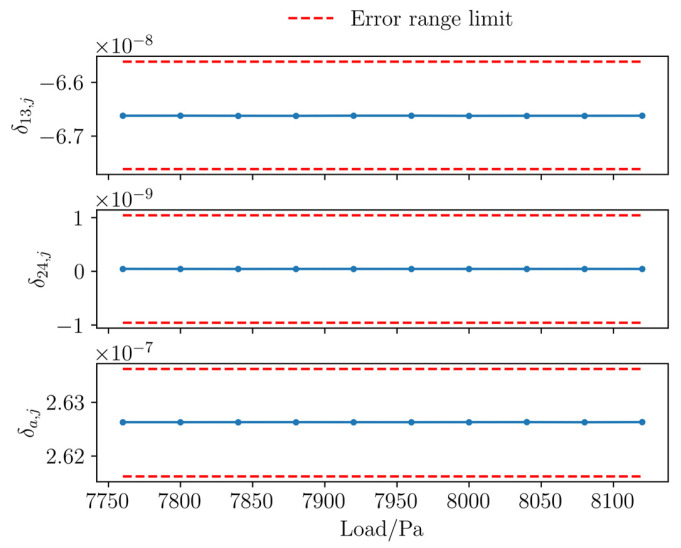
First-order difference curve of the surrogate observation values before rock fracture.

To determine the strain response index, the error-bound c must be specified according to Equation (27). Following Zhu et al. [47], the residual borehole strain after removing environmental responses is below 10^−9^; thus, c = 10^−9^ is adopted in this study. In the numerical simulation, the median of the first-order differences of the surrogate observations at 10 moments before fracture is calculated and taken as the values of *k*_13_, *k*_24_, and *k*_a_ in Equation (27) to determine the error range of each sequence and verify that the first-order difference values at these 10 moments fall within each error range. Verification of each set of data (e.g., as shown in Figure 11) proves that the numerical simulation in this study can meet the required accuracy.

**Figure 12:**
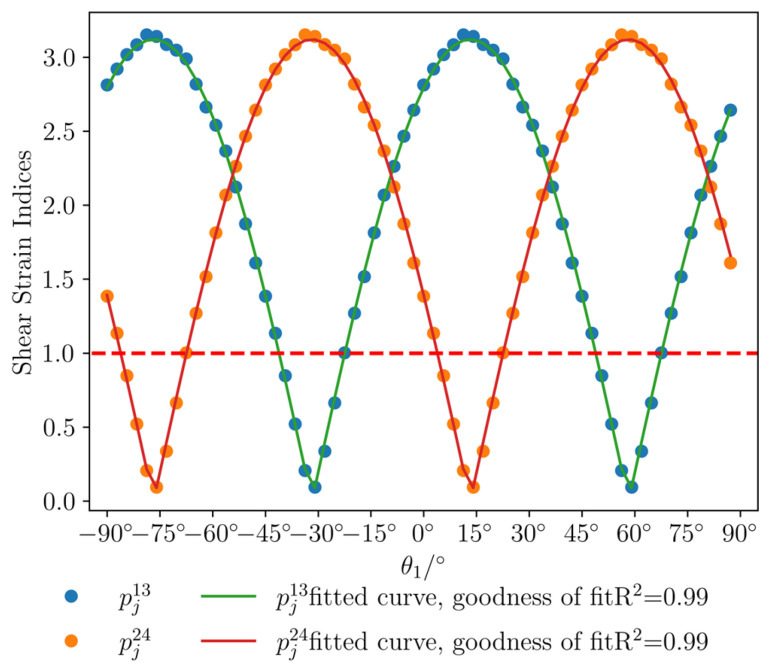
Curves of shear strain indices at different installation angles for the same borehole.

The calculated values of *k*_13_, *k*_24_, and *k*_a_ are substituted into Equation (27) to obtain the strain response indices for different installation angles of the FCBSG in the same borehole, and the strain response index curves are plotted. As shown in Figure 12, when one shear strain index reaches the maximum value, the other is the minimum value; when one shear strain index is greater than the value when the two shear strain indices are equal, the other shear strain index is less than the value when the two shear strain indices are equal. The curve characteristics are consistent with the conclusions in Section 4.1. Fitting the curves with Equation (38) yields a goodness of fit of 0.99 for all, indicating the correctness of the theoretical model. The formula for calculating the strain response indices is:

(47) $$\left\{\begin{array}{c} p_{j}^{a}=\frac{1}{c}\left|DS_{a,j}-\text{Median}(DS_{a,j-10},DS_{a,j-9},\ldots ,DS_{a,j-1})\right|\\ p_{j}^{13}=\frac{1}{\sqrt{2}c}\left|DS_{13,j}-\text{Median}(DS_{13,j-10},DS_{13,j-9},\ldots ,DS_{13,j-1})\right|\\ p_{j}^{24}=\frac{1}{\sqrt{2}c}\left|DS_{24,j}-\text{Median}(DS_{24,j-10},DS_{24,j-9},\ldots ,DS_{24,j-1})\right| \end{array}\right.$$

where c is the error range of direct observations, taken as 10^−9^; D*S*_a,*j*_, D*S*_13,*j*_, and D*S*_24,*j*_ are the first-order differences of the surrogate observations at time *j*.

For certain installation angles of the FCBSG (as shown in Figure 12), one of the shear strain indices is less than 1, indicating that this shear strain index cannot reflect the rock fracture event, while the other shear strain index is greater than 1, indicating that this shear strain index can reflect the rock fracture event. This illustrates that the combined use of the two shear strain indices can better reflect rock fracture events.

### 4.3. Influences of Distance and Direction on Strain Response Indices

To study the influence laws of distance and direction on strain response indices, the distance ρ between the borehole position and the propagating crack is set to 5 m, 10 m, 15 m, 20 m, 25 m, 30 m, 35 m, 40 m, 45 m, 50 m, 55 m, 60 m, 65 m, and 70 m, and the angle *β* between the line connecting the borehole and the center of the propagating crack and the propagating crack is set to 0°, 15°, 30°, 45°, 60°, 75°, and 90°, resulting in a total of 98 sets of numerical simulations.

Data from the 98 sets of numerical simulation results are exported. The same method as in Section 4.2 is used to verify that the first-order difference curves before fracture meet the accuracy requirements, and the FCBS response indices $p_{j}^{13}$, $p_{j}^{24}$, and $p_{j}^{a}$ of the at different installation directions in the same borehole are calculated. The obtained strain response indices are fitted using Equation (37) (assuming no cement and sleeve, A_1_ = 1/E, B_1_ = 2/E) to calculate λ_1_, λ_2_, and λ_3_, which are then substituted into Equation (45) to obtain the characteristic value of the shear strain index $p_{j}^{s}$, representing the larger one of the two shear strain indices for any installation angle of the FCBSG in the borehole at the same position.

To analyze the sensitivity of the present numerical simulation to model size and boundary conditions, additional models with dimensions of 175 m × 350 m, 150 m × 300 m, and 125 m × 250 m were constructed. Comparisons were conducted for $p_{j}^{a}$ and $p_{j}^{s}$ around the borehole with *ρ* = 5 m and *β* = 0°, and the difference between $p_{j}^{a}$ and $p_{j}^{s}$ was found to be no more than 1%. Similarly, loading types, including concentrated load and partially uniform load, were adopted, and comparisons of $p_{j}^{a}$ and $p_{j}^{s}$ were performed for the borehole with *ρ* = 5 m and *β* = 0°. The difference between $p_{j}^{a}$ and $p_{j}^{s}$ was less than 5%, demonstrating the rationality of the present numerical model for investigating borehole strain.

**Figure 13:**
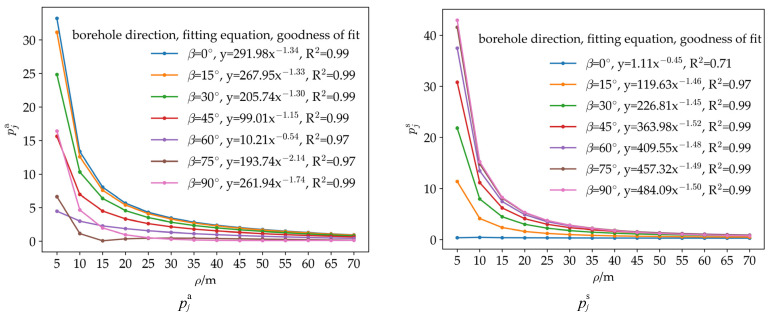
Curves of the areal strain index $p_{j}^{a}$ and the characteristic value of the shear strain index $p_{j}^{s}$ with the distance ρ from the borehole to the propagating crack based on the criterion that an areal strain index greater than 1 indicates that the index can reflect rock fracture events. A characteristic value of the shear strain index greater than 1 indicates that the larger shear strain index of the two shear strain indices can reflect rock fracture events, the effective ranges of rock fracture reflection by the areal strain index and the shear strain index are determined, and curves of the effective distances of rock fracture reflection by the areal strain index and the characteristic value of the shear strain index for different orientation of the borehole are plotted. As shown in Figure 14, when the orientation of the borehole *β* = 0°, the effective distance of rock fracture reflection by the areal strain index $p_{j}^{a}$ is 65 m, while the effective distance of rock fracture reflection by the characteristic value of the shear strain index is 0 m, with a difference of 65 m between the two; when *β* = 75°, the effective distance of rock fracture reflection by $p_{j}^{a}$ is 10 m, while the effective distance of rock fracture reflection by the characteristic value of the shear strain index is 60 m, with a difference of 50 m between the two. These numerical simulation results indicate that in certain directions of the borehole, there can be a significant difference between the effective distance of rock fracture reflection by the shear strain index and that by the areal strain index, highlighting the importance of the combined use of shear strain indices and the areal strain index.

The results of the 98 sets of simulations are plotted as shown in Figure 13. It can be seen from the figure that the areal strain index $p_{j}^{a}$ and the characteristic value of the shear strain index $p_{j}^{s}$ decrease with the increase in the distance ρ between the borehole and the fracture zone, following the variation law of the function y = ax^b^ with a negative exponent. There are significant differences in $p_{j}^{a}$ and $p_{j}^{s}$ among different orientations of the borehole *β*. The maximum value of the fitting function parameter a for the areal strain index $p_{j}^{a}$ is 291.98, and the minimum value is 10.21; the maximum value of the fitting function parameter a for the characteristic value of the shear strain index $p_{j}^{s}$ is 484.09, and the minimum value is 1.11. $p_{j}^{a}$ and $p_{j}^{s}$ in the same orientation of the borehole β show a certain degree of complementarity: when the fitting function parameter a of $p_{j}^{a}$ is 99.01, the fitting function parameter a of $p_{j}^{s}$ is 363.98; when the fitting function parameter a of $p_{j}^{a}$ is 10.21, the fitting function parameter a of $p_{j}^{s}$ is 409.55; when the fitting function parameter a of $p_{j}^{s}$ is 1.11, the fitting function parameter a of $p_{j}^{a}$ is 291.98; and when the fitting function parameter a of $p_{j}^{s}$ is 119.63, the fitting function parameter a of $p_{j}^{a}$ is 267.95. Within the range of *β* from 0° to 90°, the maximum value of the fitting function parameters a of $p_{j}^{a}$ and $p_{j}^{s}$ is greater than 200; $p_{j}^{a}$ first decreases and then increases with the change in the orientation of the borehole *β* from 0° to 90°, while $p_{j}^{s}$ continuously increases with the change in *β* from 0° to 90°.

**Figure 14:**
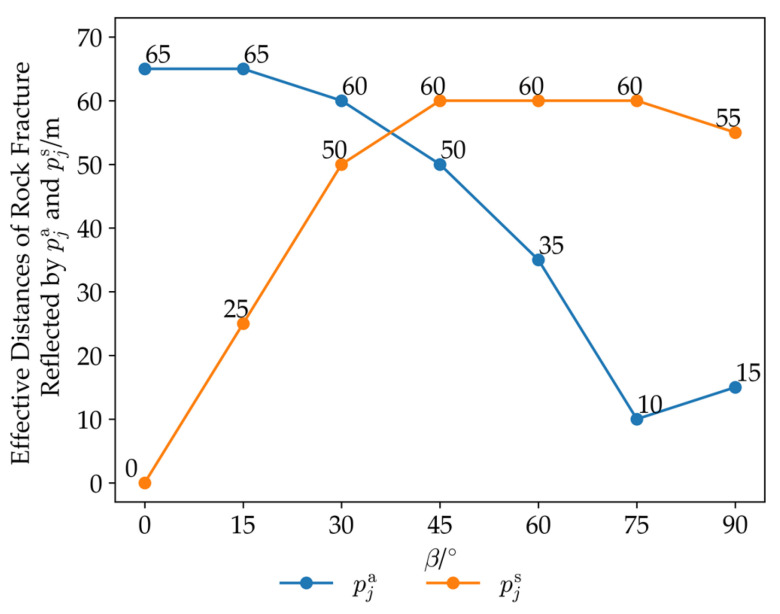
Effective distances of rock fracture reflected by $p_{j}^{a}$ and $p_{j}^{s}$ under different orientations of the borehole.

Among the 98 sets of numerical simulations, the areal strain index alone can reflect rock fracture events in 60 sets, the shear strain index alone can reflect rock fracture events in 62 sets, and the combined use of the areal strain index and the shear strain index can reflect rock fracture events in 85 sets. Within the range of *β* = 0° to *β* = 90°, the minimum range of rock fracture that can be reflected by the three strain response indices is 55 m, the maximum range is 65 m, and the average range is 60.7 m.

## 5. Discussion

This study adopts the linear elasticity assumption combined with the cohesive zone model (CZM), which is mainly applicable to the monitoring of fracture propagation in brittle or quasi-brittle rock media. The model assumes that the rock matrix behaves in a linear elastic manner, with softening occurring only at the crack tip. Therefore, the conclusions of this paper can explain the effective monitoring of three strain response indicators based on borehole strain for hard rocks such as tight sandstone and carbonate rock, as well as the variation laws of the three strain response indicators with borehole installation angle, rock fracture distance, and orientation of the borehole. However, for borehole strain monitoring of soft rocks, salt rocks, or rock masses that exhibit significant plastic flow and large deformation characteristics under high stress, the research results of this paper fail to clarify their monitoring effectiveness and variation laws.

Based on the static load assumption, this study defines its scope of application mainly for working conditions involving quasi-static fracture propagation or slow extension. Due to the neglect of dynamic effects and inertial forces, this study is only applicable to the process of stable crack opening and slow growth under steady external loads. It is not applicable to working conditions accompanied by significant dynamic effects, such as instantaneous crack initiation, high-speed slip, or unstable fracture propagation.

This study neglects the effect of the cement sheath and assumes an ideal geometric shape of the fracture surface. It is applicable to macro-scale fracture propagation monitoring and scenarios with idealized cementing quality, but not to cases with severe defects in cement sheath bonding, or complex inhomogeneous deformation caused by fracture surface roughness and tortuosity.

The research results of this study, using three indicators to characterize fracture propagation, are determined based on the error range of the YRY-4 strain gauge after removing environmental noise. Both the judgment of fracture propagation state and the setting of indicator thresholds in this study take this accuracy level as the physical benchmark. Therefore, in practical engineering, the indicators proposed in this paper can only be used for predicting rock fracture after using the YRY-4 strain gauge and removing environmental noise.

## 6. Conclusions

Through numerical simulation methods, this study takes FCBSG as the monitoring carrier, explores the FCBS response characteristics during large-scale local rock fracture, and proposes strain response indices to describe such response characteristics. The main conclusions are as follows:

(1) For strain response indices at different installation angles in the same borehole, the areal strain index is not affected by the installation direction of the FCBSG and maintains consistency. The shear strain indices show regular changes—one of the two shear strain indices is not less than the characteristic value of the shear strain index, and the other is less than or equal to it, with the maximum value of the shear strain index equal to $\sqrt{2}/2$ times the characteristic value of the shear strain index. This law indicates that regardless of the installation direction of the FCBSG, at least one shear strain index can maintain high monitoring sensitivity.
(2) Influences of rock fracture distance and orientation of the borehole on strain response indices. The larger values of the areal strain index and shear strain index decrease with the increase in the distance ρ between the borehole and the fracture zone, following the variation law of the function y = ax^b^ with a negative exponent. There are significant differences in the larger values of the areal strain index and shear strain index among different orientation of the borehole *β*: the maximum value of the fitting function parameter a for the areal strain index $p_{j}^{a}$ is 291.98, and the minimum value is 10.21; the maximum value of the fitting function parameter a for the characteristic value of the shear strain index $p_{j}^{s}$ is 484.09, and the minimum value is 1.11. The larger values of the areal strain index and shear strain index in the same orientation of the borehole *β* show a certain degree of complementarity: within the range of β from 0° to 90°, the maximum value of the fitting function parameters a of $p_{j}^{a}$ and $p_{j}^{s}$ is greater than 200.
(3) In certain directions of the borehole, there can be a significant difference between the effective distance of rock fracture reflection by the larger shear strain index and that by the areal strain index. When the orientation of the borehole *β* = 0°, the difference between the effective distances of rock fracture reflection by the larger shear strain index and the areal strain index is 65 m; when *β* = 75°, the difference is 50 m. This indicates that the combined use of shear strain indices and the areal strain index can better reflect rock fracture events.
(4) Within the range of *β* = 0° to *β* = 90°, the minimum range of rock fracture that can be reflected by the three strain response indices is 55 m, the maximum range is 65 m, and the average range is 60.7 m.

## Figures and Tables

**Table 1:** Physical and mechanical parameters.

E	**μ**	$\sigma_{\text{max}}^{o}$	$\delta_{n}^{f}$
35 GPa	0.25	3.5 MPa	0.01 mm

## Data Availability

The raw data supporting the conclusions of this article will be made available by the authors on request.
